# Age Differences in Vestibular Brain Connectivity Are Associated With Balance Performance

**DOI:** 10.3389/fnagi.2020.566331

**Published:** 2020-11-16

**Authors:** Fatemeh Noohi, Catherine Kinnaird, Yiri De Dios, Igor S. Kofman, Scott J. Wood, Jacob Bloomberg, Ajitkumar Mulavara, Kathleen H. Sienko, Thad A. Polk, Rachael D. Seidler

**Affiliations:** ^1^Department of Kinesiology, University of Michigan, Ann Arbor, MI, United States; ^2^Department of Psychology, University of Michigan, Ann Arbor, MI, United States; ^3^Department of Biomedical Engineering, University of Michigan, Ann Arbor, MI, United States; ^4^KBR, Houston, TX, United States; ^5^NASA Johnson Space Center, Houston, TX, United States; ^6^Department of Applied Physiology and Kinesiology, University of Florida, Gainesville, FL, United States

**Keywords:** aging, balance performance, functional connectivity, sensory weighting, vestibular system

## Abstract

Visual and auditory brain network connectivity decline with age, but less is known about age effects on vestibular functional connectivity and its association with behavior. We assessed age differences in the connectivity of the vestibular cortex with other sensory brain regions, both during rest and during vestibular stimulation. We then assessed the relationship between vestibular connectivity and postural stability. A sample of seventeen young and fifteen older adults participated in our study. We assessed the amount of body sway in performing the Romberg balance task, with degraded somatosensory and visual inputs. The results showed no significant difference in balance performance between age groups. However, functional connectivity analyses revealed a main effect of age and condition, suggesting that vestibular connectivity was higher in young adults than older adults, and vestibular connectivity increased from resting state to stimulation trials. Surprisingly, young adults who exhibited higher connectivity during stimulation also had greater body sway. This suggests that young adults who exhibit better balance are those who respond more selectively to vestibular inputs. This correlation is non-significant in older adults, suggesting that the relationship between vestibular functional connectivity and postural stability differs with age.

## Introduction

In humans, the brain’s vestibular network comprises multiple cortical and subcortical regions that communicate with each other to process vestibular inputs. These regions include (but are not limited to) the parieto-insular vestibular cortex (PIVC), the vestibular nuclei, the thalamus and the cerebellum. Many studies have looked at the functional and anatomical connectivity of these regions using fMRI and diffusion MRI (dMRI) techniques. Kirsch et al. (2016) provided evidence that bilateral vestibular nuclei and bilateral insular cortex are both functionally and anatomically connected. Eickhoff et al. (2010) also reported distinct connectivity patterns for core vestibular cortical regions. The operculum parietale (OP) showed functional and anatomical connectivity with regions of higher order somatosensory processing (anterior inferior parietal cortex, intraparietal sulcus, ventroposterior lateral and inferior nuclei of thalamus). Although Eickhoff et al.’s (2010) results were generally consistent with a congruency between functional and anatomical connectivity maps, there were also some discrepancies, which could reflect the fact that co-activation of brain regions can occur independently of their anatomical connections. Also, in their previous work (Eickhoff et al., 2006), they found no anatomical asymmetry between left and right OP, despite the fact that there was significant functional asymmetry in vestibular processing. More recently, zu Eulenburg et al. (2012) conducted a meta-analysis suggesting that OP was the core region of the vestibular network. They further implemented a connectivity analysis which revealed functional connectivity of right OP with temporo-parietal cortex, premotor cortex, and the midcingulate gyrus.

To the best of our knowledge, only one study has investigated the effects of healthy aging on vestibular network connectivity. Cyran et al. (2016) reported age differences in functional connectivity of the vestibular network during galvanic vestibular stimulation. They found that vestibular network connectivity was reduced in older adults, whereas the somatosensory network showed no functional connectivity age differences. Moreover, they showed that these age differences in vestibular network functional connectivity were independent of age-related structural alterations and were associated with declines in magnitude of the blood oxygen level-dependent (BOLD) response. They did not conduct any balance assessments to determine the functional consequences of these age differences in vestibular connectivity, however.

Age-related declines in functional connectivity have also been reported in other networks outside the vestibular system. For example, Cassady et al. (2019) reported that the sensorimotor network became less segregated from other functional networks with increasing age. Roski et al. (2013) reported that older adults had reduced resting state sensorimotor and visual network connectivity. In a study of age effects on auditory cortex functional connectivity, Peelle et al. (2010) found that older adults exhibit less activity in speech comprehension core regions and reduced task-based connectivity between these areas. Mowinckel et al. (2012) found reduced resting state connectivity within the visual and default mode networks for older versus young adults. Yang et al. (2014) also showed that with advancing age, the precuneus network merged with the default mode network, and specific functional connectivity patterns became less distinguishable between the two. Some studies suggest that age-related changes in functional connectivity could be more heterogeneous. For example, Tomasi and Volkow (2012) showed that age-related alterations in resting state functional connectivity varied based on the network. The default mode network and dorsal attention network showed age-related decline in functional connectivity, while the somatosensory and subcortical networks showed age-related increase in connectivity. Thus, we hypothesized that younger individuals would exhibit overall greater vestibular connectivity compared to older adults (during both the rest and vestibular stimulation), which could vary depending on the network.

Greater functional connectivity has often been associated with better behavior performance, including better motor performance. For example, Yuan et al. (2015) found that greater resting state functional connectivity (between sensorimotor, visual, vestibular, and left fronto-parietal cortical areas) was associated with higher walking speed in older adults. Pleger et al. (2006) applied excitatory repetitive Transcranial Magnetic Stimulation (rTMS) to primary sensory cortex and induced increased connectivity from the somatosensory to primary motor cortex, which in turn was associated with behavioral improvements in tactile discrimination. Based on these findings, we hypothesized that greater vestibular connectivity would be associated with better balance.

## Materials and Methods

### Participants

We recruited 32 healthy adults (15 older: 65–80, $\bar{x}$ = 71.2 ± 4.14, 10 females and 18 young: 18–35, $\bar{x}$ = 21 ± 2.44, 8 females) from the University of Michigan clinical studies website. All subjects were right-handed. The exclusion criteria were defined as any vestibular, neurological, auditory, or postural disorders. In addition to the Montreal Cognitive Assessment (MOCA), a typical MRI screening was conducted. The University of Michigan Institutional Review Board approved the study, and all subjects signed the consent form prior to participation.

### Balance Assessments

In this study, we focused on the subjects’ performance in the Romberg stance task (feet together), on a compliant surface with eyes closed and arms crossed. In this task visual input is removed, and somatosensory inputs are degraded, so individual differences in vestibular processing play a large role in balance maintenance.

We calculated the amount of body sway as the area of an ellipse fit to the 95th percentile confidence interval of center of pressure motion in the anterior-posterior and medial-lateral directions (Sienko et al., 2008; Lee et al., 2012). Better postural stability was reflected as less body sway and smaller ellipse size. We instructed participants to maintain their balance for the whole duration of the trial (i.e., 30 s). Each participant was given one trial to perform the task to avoid the confound of practice effect, and the trial was stopped if participants lost their balance.

### FMRI Data Acquisition

The image acquisition was conducted on the same day as the balance assessment. Magnetic resonance images were acquired using a 3.0 Tesla MRI scanner (General Electric Medical Systems, DISCOVERY MR750). The parameters of the acquisition for structural images included a T1-weighted 3D IR Prepped FSPGR, TR = 12.2 s, TE = 5.1 ms, FA = 15°, matrix size = 256 × 192, FOV = 260 × 260 mm, slice thickness = 1 mm, and number of slices = 152. The parameters of the acquisition for functional images (both resting state and stimulation blocks) included a gradient-echo plane ascending sequence, FOV = 220 mm, TR = 2 s, TE = 30 ms, number of slices = 43, voxel size = 3.4375 × 3.4375 mm, slice thickness = 3 mm (no spacing), and FA = 90.

Head motion was restricted using a Velcro strap over the forehead. Pads were placed around the head to protect hearing (in addition to ear plugs) and to further limit head movements. To collect physiological responses (cardiac and respiration rates), a pulse oximeter was placed on the subjects’ index finger and a respirometer belt was wrapped around the abdomen.

The acquisition protocol was as follows: high-resolution T1 structural scan (SPGR), resting state scan, and vestibular stimulation scan. Subjects were instructed to keep their eyes open during the resting state scan, while keeping their gaze on a fixation point presented to them on the screen. They were instructed to close their eyes during the vestibular stimulation scan to minimize visual processing that could interact with the vestibular system. The resting state scan lasted 10 min, and the vestibular stimulation scan lasted 4 min. The vestibular stimulation was applied in the form of pneumatic skull taps as explained in our previous work (Noohi et al., 2017): This method is verified to successfully induce the ocular Vestibular Evoked Myogenic Potential (oVEMP), which objectively confirms that the skull taps lead to stimulation of the vestibular system, but not somatosensory or visual regions (in fact, these sensory regions are deactivated in response to vestibular stimulation).

The taps were delivered over the left cheekbone, in five alternating blocks of stimulation (24 s) and rest (20 s). We did not apply an individualized stimulus to account for differences in subjects’ sensory perception thresholds because the purpose of the current study was to assess the average differences between the two age groups.

## Data Analysis

### Balance Performance

We used Vicon’s Nexus software to analyze the center of pressure (COP) data that were collected at 100 Hz on the force platform. The force platform channels were plugged into a data acquisition board and the data were recorded using the Nexus software, which then automatically calculated COP data from the raw channel data. The COP data were then exported from the Nexus software and outcome measures were calculated. Similar to previous studies (Sienko et al., 2008; Lee et al., 2012), we applied a low pass filter with a 2nd order recursive Butterworth filter with a cutoff of 10 Hz. For each balance trial, we fitted a 95% confidence interval ellipse to the anterior-posterior and medial-lateral trajectories. The age group differences in the area of the ellipse were assessed using Mann-Whitney *U* test. The area of the ellipse was then implemented in correlation with individual differences in brain connectivity patterns. A subgroup of subjects (i.e., three older adults and six young adults) were dropped from the analyses due to data collection issues (e.g., subjects were unable to complete the 30 s without stepping off the compliant surface or opening their eyes) or being an outlier (>3 standard deviation).

### FMRI Analysis

We used SPM12 software (Welcome Department of Cognitive Neurology, London, United Kingdom (Friston et al., 1995)) and the CONN functional connectivity toolbox v14 (Whitfield-Gabrieli and Nieto-Castanon, 2014) to preprocess and analyze the fMRI data. The preprocessing steps were completed using the CONN toolbox default preprocessing pipeline: (1) Structural segmentation (gray matter, white matter, CSF) and normalization to the Montreal Neurological Institute (MNI152) template (Friston et al., 1995). (2) Functional realignment, slice timing correction, outlier detection using the ARTifact detection toolbox (NITRC)^1^ to detect any volumes with >2 mm translational or >2° rotational movement, segmentation (gray matter, white matter, CSF) and MNI normalization. We segmented and normalized the cerebellum separately using the Spatially Unbiased Infra-tentorial Template (SUIT, Diedrichsen, 2006; Diedrichsen et al., 2009, 2011; Diedrichsen and Zotow, 2015).

We estimated functional connectivity between regions identified in our previous work (Noohi et al., 2019) that were responsive to vestibular stimulation. These regions included the Parietal Operculum, Intracalcarine Cortex, Postcentral Gyrus, Temporal Pole, Brainstem, Cerebellar Lobule VI, and Crus I. Using Harvard-Oxford cortical and subcortical structural atlases^2^, we created a sphere (10 mm diameter) around the corresponding coordinates (5 mm diameter for brainstem), and functional connectivity data were derived from unsmoothed data to avoid spillage from neighboring voxels. For all the functional connectivity results, we report the combined average times series of the crus I and lobule VI ROIs as the cerebellum ROI, since their time courses were strongly correlated with each other (*r* > 0.9).

An aCompCor denoising procedure was applied to remove the effects of confounding factors (white matter, CSF, motion, and main task effects). A band pass filter threshold of [0.008 Inf] was applied to both resting state and stimulation runs. A first level analysis was then performed using a General Linear Model (GLM) for the resting state data and a general Psycho-Physiologic Interaction (gPPI) model for stimulation blocks. The bivariate correlation measures resulting from the GLM represented the connectivity during rest (e.g., a positive correlation indicated a positive association between the time series of two ROIs during rest, and a negative correlation represented an anti-correlation between the time series of two ROIs during rest). The bivariate correlation measures resulting from the gPPI analysis indicated the change in connectivity during stimulation compared to an implicit baseline condition (i.e., rest blocks). Thus, positive correlations reflected higher connectivity during stimulation relative to baseline and negative correlations reflected lower connectivity during stimulation relative to baseline.

Next, a ROI to ROI analysis was performed to quantify connectivity. The correlation between each ROI and all of the others was calculated during the rest and vestibular tap stimulation conditions. We applied the false discovery rate (FDR) error correction to correct for multiple comparisons (Chumbley et al., 2010). The results were considered significant if they reach the *p* < 0.05 (FDR) at the peak voxel level. We conducted the analysis for resting state and stimulation runs separately at the first level, and then analyzed the alterations and age differences in the connectivity pattern using *t*-tests at the second level.

We extracted the average time series of all voxels within each ROI and conducted correlation analyses to compare the average time series of selected ROIs during rest and stimulation. We selected the parietal operculum (i.e., vestibular cortex) as the seed ROI and compared its average time series with the other ROIs. Positive connectivity of the vestibular cortex with any of the other ROIs suggests a synchronous pattern of their time series (i.e., activation and deactivation at similar times) and does not necessarily reflect an overall increase in the BOLD signal. The correlation coefficients were standardized (i.e., using Fisher’s R to Z transformation) before entering to second level analyses. The multivariate ANOVA was applied to assess age by ROI by condition interaction. Lastly, we assessed the relationship between overall vestibular connectivity and individual differences in age and balance. For this analysis, we calculated the vestibular connectivity with all ROIs using Principle Component Analysis and implementing the first eigenvariate in correlation with COP measures. We conducted all correlation analyses using Spearman method, with confidence intervals bootstrapped with 1000 permutations. The correction for multiple comparisons was conducted using the Benjamini-Hochberg procedure with a %5 false discovery rate (Benjamini and Hochberg, 1995; Lindquist and Mejia, 2015).

## Results

### Balance Assessment

The CoP measures were not significantly different between young and older adults in performing the Romberg task (*W* = 328, *p* = 0.16).

### Functional Connectivity

**FIGURE 1:**
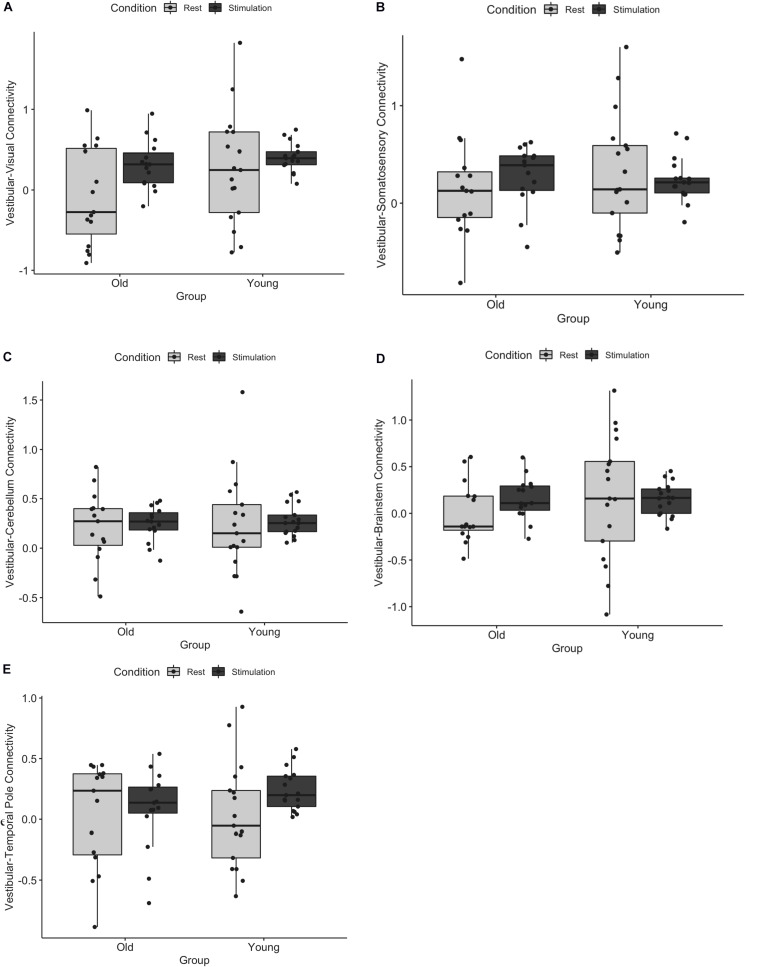
Functional connectivity of vestibular cortex with **(A)** visual, **(B)** somatosensory, **(C)** cerebellum, **(D)** brainstem, and **(E)** temporal pole during rest and tap stimulation is shown for young and older adults. There was a significant main effect of age, a main effect of condition, and an age by condition by ROI interaction. Error bars represent standard deviation.

**FIGURE 2:**
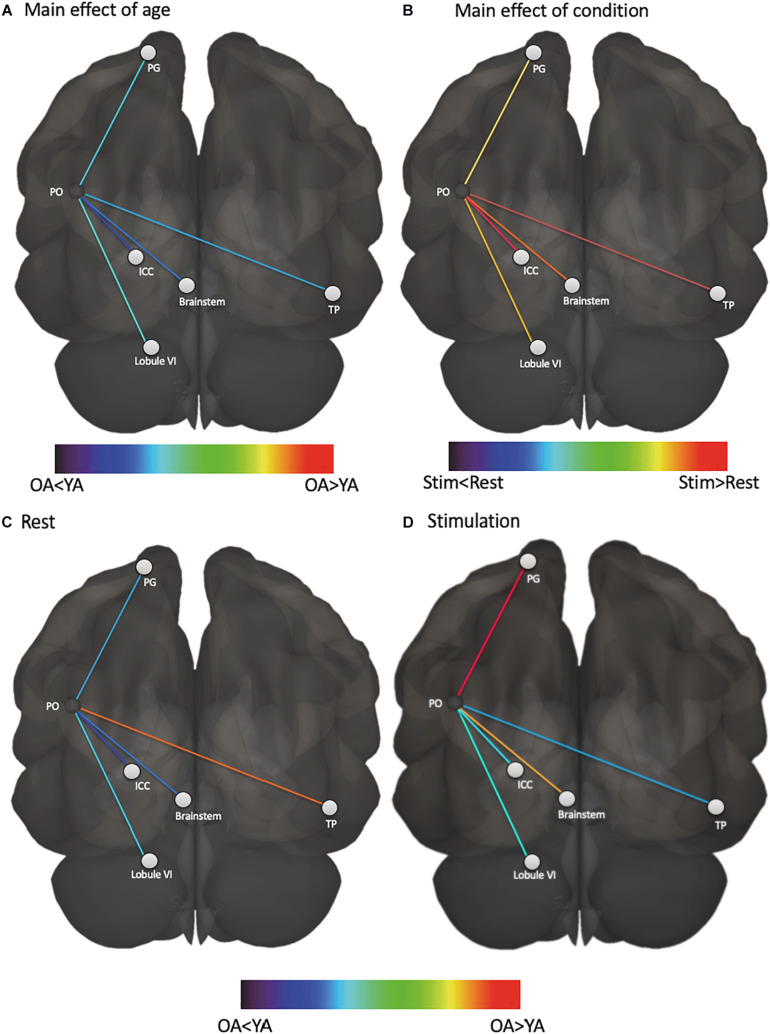
Functional connectivity of vestibular cortex varies based on age and condition. **(A)** vestibular functional connectivity was higher in young adults than older adults, regardless of condition, **(B)** vestibular functional connectivity was higher during stimulation than rest, regardless of age, **(C)** vestibular functional connectivity with temporal pole was higher in older adults at rest compared to young adults at rest, and **(D)** vestibular functional connectivity with somatosensory cortex and brainstem was higher in older adults during stimulation compared to young adults during stimulation. PO, parietal operculum; PG, postcentral gyrus; ICC, intra-calcarine cortex; TP, temporal pole; YA, young adults; OA, older adults.

We first assessed whether functional connectivity of the vestibular cortex changed from rest to stimulation, and whether this effect differed for young and older adults. A three-way ANOVA of age, ROI, and condition (i.e., rest vs. stimulation) showed a significant main effect of condition (i.e., connectivity was higher during vestibular stimulation relative to rest, *F* = 14.84, *p* = 0.001), a main effect of age (i.e., young adults exhibited overall greater connectivity than older adults, *F* = 3.91, *p* = 0.04), and an age by condition by ROI interaction (*F* = 2.33, *p* = 0.04) (Figure 1). This interaction appears to be driven by an increase in vestibular-temporal pole connectivity from rest to stimulation in young adults, although this effect did not survive the Benjamini-Hochberg correction for multiple comparisons. A brain map illustration of these results is shown in Figure 2.

### Functional Connectivity’s Association With Balance

**FIGURE 3:**
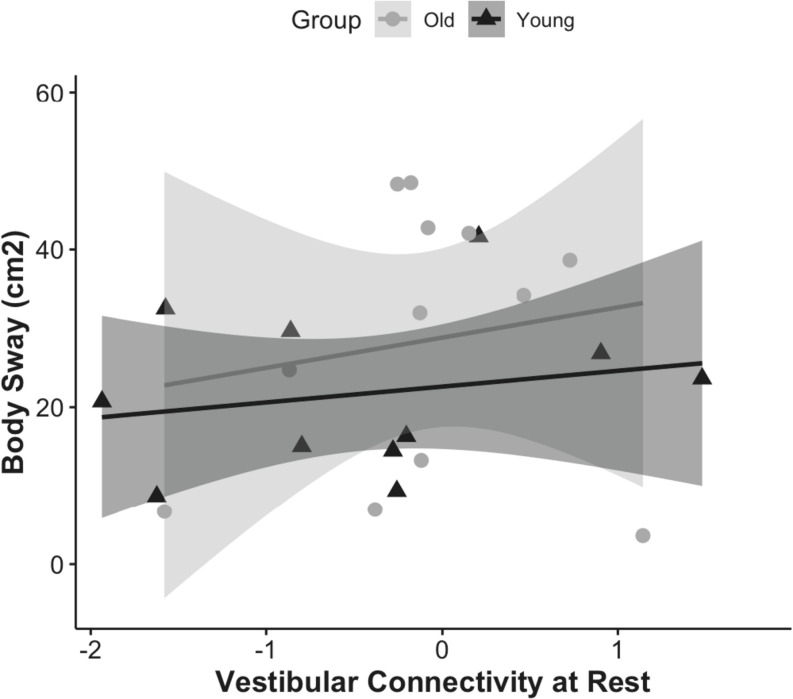
Correlation of vestibular connectivity at rest with balance performance is shown for young and older adults as rho = 0.23, *p* = 0.4, CI (0, 0.31) and rho = −0.09, *p* = 0.7, CI (0, 0.28); respectively. The figure represents the subset of each group who completed the balance task, corrected for outliers.

First, we examined whether the resting state functional connectivity of the vestibular cortex was associated with postural stability, and whether this effect was modulated by age (Figure 3). The overall resting state functional connectivity of vestibular cortex did not associate with postural stability in young or older adults.

Next, we assessed whether functional connectivity of the vestibular cortex during vestibular stimulation was associated with postural control, and whether this effect was modulated by age.

**FIGURE 4:**
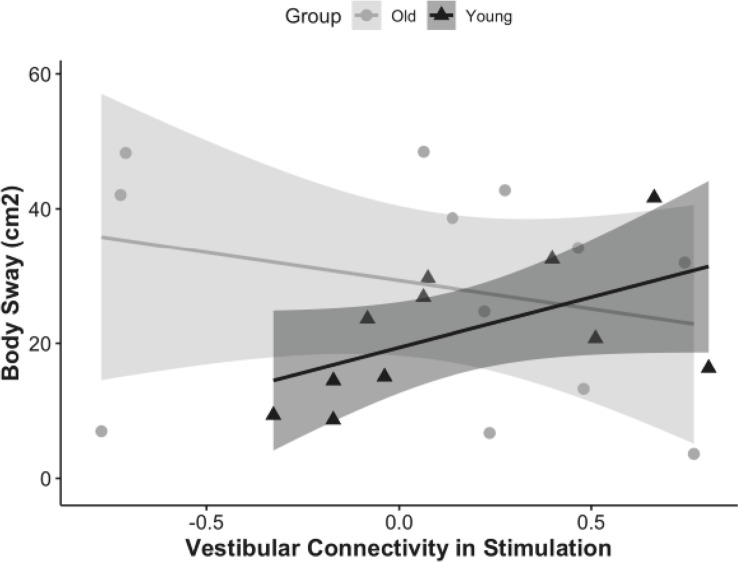
Correlation of vestibular connectivity during stimulation with balance performance is shown for young and older adults as rho = 0.68, *p* = 0.02, CI (0, 0.84) and rho = –0.41, *p* = 0.1, CI (0, 0.49); respectively. The figure represents the subset of each group who completed the balance task, corrected for outliers.

The results showed that overall functional connectivity of vestibular cortex during stimulation was associated with postural stability in young adults (rho = 0.78, *p* = 0.02, CI = 0, 0.84), but not in older adults (rho = −0.41, *p* = 0.1, CI = 0, 0.49) (Figure 4). Interestingly, higher connectivity between the vestibular cortex and all the other regions was associated with a higher amount of body sway (i.e., worse postural stability). We followed up by analyzing the relationship between body sway and connectivity to each of the ROIs individually. Higher vestibular-somatosensory connectivity was associated with more body sway (rho = 0.60, *p* = 0.05, CI = 0.04, 0.92).

## Discussion

In this study we investigated age differences in connectivity of the vestibular cortex with other sensorimotor regions, previously identified to be involved in processing vestibular information. We assessed vestibular connectivity in the resting state and during vestibular stimulation. Lastly, we examined the relationship between individuals’ balance and their functional connectivity patterns.

Our findings are in general agreement with previous studies suggesting that older adults exhibit an overall decline in functional connectivity compared to young (Peelle et al., 2010; Mowinckel et al., 2012; Roski et al., 2013; Yang et al., 2014; Cyran et al., 2016). We found that regardless of condition (i.e., rest or stimulation), young adults generally exhibited greater connectivity than older adults. This suggests a distinction between how young and older adults modulate vestibular brain regions, with the young exhibiting more synchronous neural responses between vestibular cortex and other sensorimotor brain regions.

By including the balance metrics, we were able to interpret the functional consequences of the observed age differences in vestibular connectivity. Some studies suggest that higher resting state functional connectivity in older adults is associated with better motor performance. Findings of Langan et al. (2010) suggested that reduced resting state interhemispheric connectivity was associated with over-recruitment of the non-dominant hemisphere during motor task performance for older adults, which was correlated with longer reaction times during motor performance. Similarly, Seidler et al. (2015) showed that greater resting state connectivity between motor cortex, putamen, insula and cerebellum was associated with better motor performance in older adults. Contrary to these reports, we did not find a significant correlation between greater resting-state connectivity of vestibular cortex and older adults’ balance performance. Moreover, there was largely no significant age difference in correlation of balance with resting state vestibular connectivity (i.e., vestibular connectivity at rest was not associated with balance performance for either young or older adults).

Unlike in the resting state, vestibular connectivity during stimulation was correlated with individuals’ balance performance, and this correlation varied based on age. Those young adults who engaged the vestibular cortex more selectively (i.e., they had less vestibular connectivity with other ROIs) were the ones who performed better. This doesn’t seem to be the case for older adults; those who exhibited more involvement of other regions (i.e., more vestibular connectivity with other ROIs) showed a trend for better performance. This could be related to older adults having altered connectivity with regions outside of our selected ROIs (e.g., frontal cortex). The fact that the association between vestibular connectivity and balance was only evident in young adults could be interpreted in light of the compensation-related utilization of neural circuits hypothesis (CRUNCH), proposed by Reuter-Lorenz and Cappell (2008). According to this theory, older adults can compensate for their inefficient neural processing by engaging more brain regions compared to the young. As the current findings show, to perform a high demanding balance task the older adults relied on recruiting additional neural resources, and therefore, exhibited higher vestibular connectivity when performing better. However, the performance in the Romberg task (i.e., standing with feet together, on a compliant surface, with eyes closed and arms crossed) is inherently reliant on the brain’s ability to reweight the allocation of neural resources in favor of vestibular processing. The utility of this compensation strategy is demonstrated by the rather surprising finding that there were no differences in the posture measures between young and old groups.

Many studies have used non-invasive brain stimulation methods to modulate functional connectivity of different brain regions. For example, Cai et al. (2018) used galvanic vestibular stimulation to enhance the deficient connectivity of pedunculopontine nucleus in Parkinson’s disease patients. The brain regions identified in the present study could be potential targets of brain stimulation to modulate the functional connectivity patterns of older adult bad performers and make them more similar to older adult good performers. The behavioral outcomes of such neural modulation could be especially beneficial for older adults who cannot benefit from conventional balance training interventions.

## Limitations

The main limitation of the current study is the small sample size and lack of power. Also, the COP measures could be improved by controlling for confounding factors such as height and body mass index. Using individualized stimulation thresholds for each participant would help to minimize the potential role of peripheral vestibular declines (e.g., inner ear).

Further, the older adults who participated in this study represent a relatively high functioning population; thus, future studies are needed to verify the current findings in a larger sample of healthy older adults.

## Conclusion

In this study we addressed two questions: does vestibular connectivity decline with age? And does it associate with balance/stability? We found that young adults exhibited greater vestibular connectivity than older adults, and that their vestibular connectivity was related to their balance performance. Those young adults who exhibited better balance were those who responded more selectively to vestibular inputs (i.e., lower vestibular connectivity during stimulation). This pattern was not observed in older adults, suggesting that the correlation between vestibular functional connectivity and postural stability varies by age.

## Data Availability Statement

The raw data supporting the conclusions of this article will be made available by the authors, without undue reservation.

## Ethics Statement

The studies involving human participants were reviewed and approved by the University of Michigan Institutional Review Board. The patients/participants provided their written informed consent to participate in this study.

## Author Contributions

SW, JB, AM, and RS: conceptualization. FN and CK: data curation and formal analysis. RS: funding acquisition. YD, IK, KS, and RS: methodology. FN, CK, and YD: project administration. KS and RS: resources. TP and RS: supervision. FN: writing – original draft. FN, CK, YD, IK, SW, JB, AM, KS, TP, and RS: writing – review and editing. All authors contributed to the article and approved the submitted version.

## Conflict of Interest

YD was employed by company KBR. The remaining authors declare that the research was conducted in the absence of any commercial or financial relationships that could be construed as a potential conflict of interest.

## Footnotes

1 *Artifact Detection Tools (ART)*. Available online at: https://www.nitrc.org/projects/artifact_detect/

2 https://identifiers.org/neurovault.collection:262

## References

[B1] Benjamini Y. Hochberg Y. (1995). Controlling the false discovery rate: a practical and powerful approach to multiple testing. *J. R. Stat. Soc. Ser. B* 57 289–300. 10.1111/j.2517-6161.1995.tb02031.x

[B2] Cai J. Lee S. Ba F. Garg S. Kim L. J. Liu A. (2018). Galvanic vestibular stimulation (GVS) augments deficient pedunculopontine nucleus (PPN) connectivity in mild Parkinson’s Disease: fMRI effects of different stimuli. *Front. Neurosci.* 12:101. 10.3389/fnins.2018.00101 PMC5835530 29541016

[B3] Cassady K. Gagnon H. Lalwani P. Simmonite M. Foerster B. Park D. (2019). Sensorimotor network segregation declines with age and is linked to GABA and to sensorimotor performance. *Neuroimage* 186, 234–244. 10.1016/j.neuroimage.2018.11.008 30414983 PMC6338503

[B4] Chumbley J. Worsley K. Flandin G. Friston K. (2010). Topological FDR for neuroimaging. *Neuroimage* 49 3057–3064. 19944173 10.1016/j.neuroimage.2009.10.090 PMC3221040

[B5] Cyran C. A. M. Boegle R. Stephan T. Dieterich M. Glasauer S. (2016). Age-related decline in functional connectivity of the vestibular cortical network. *Brain Struct. Funct.* 221 1443–1463. 10.1007/s00429-014-0983-6 25567421

[B6] Diedrichsen J. (2006). A spatially unbiased atlas template of the human cerebellum. *Neuroimage* 33 127–138. 10.1016/j.neuroimage.2006.05.056 16904911

[B7] Diedrichsen J. Balsters J. H. Flavell J. Cussans E. Ramnani N. (2009). A probabilistic MR atlas of the human cerebellum. *Neuroimage* 46 39–46. 10.1016/j.neuroimage.2009.01.045 19457380

[B8] Diedrichsen J. Maderwald S. Küper M. Thürling M. Rabe K. Gizewski E. R. (2011). Imaging the deep cerebellar nuclei: a probabilistic atlas and normalization procedure. *Neuroimage* 54 1786–1794. 10.1016/j.neuroimage.2010.10.035 20965257

[B9] Diedrichsen J. Zotow E. (2015). Surface-based display of volume-averaged cerebellar imaging data. *PLoS One* 10:e0133402. 10.1371/journal.pone.0133402 PMC4521932 26230510

[B10] Eickhoff S. B. Jbabdi S. Caspers S. Laird A. R. Fox P. T. Zilles K. (2010). Anatomical and functional connectivity of cytoarchitectonic areas within the human parietal operculum. *J. Neurosci.* 30 6409–6421. 10.1523/jneurosci.5664-09.2010 20445067 PMC4791040

[B11] Eickhoff S. B. Weiss P. H. Amunts K. Fink G. R. Zilles K. (2006). Identifying human parieto-insular vestibular cortex using fMRI and cytoarchitectonic mapping. *Hum. Brain Mapp.* 27 611–621. 10.1002/hbm.20205 16281284 PMC6871353

[B12] Friston K. J. Ashburner J. Frith C. D. Poline J.-B. Heather J. D. Frackowiak R. S. J. (1995). Spatial registration and normalization of images. *Hum. Brain Mapp.* 3 165–189. 10.1002/hbm.460030303

[B13] Kirsch V. Keeser D. Hergenroeder T. Erat O. Ertl-Wagner B. Brandt T. (2016). Structural and functional connectivity mapping of the vestibular circuitry from human brainstem to cortex. *Brain Struct. Funct.* 221 1291–1308. 10.1007/s00429-014-0971-x 25552315

[B14] Langan J. Peltier S. J. Bo J. Fling B. W. Welsh R. C. Seidler R. D. (2010). Functional implications of age differences in motor system connectivity. *Front. Syst. Neurosci.* 4:17. 10.3389/fnsys.2010.00017 20589101 PMC2893009

[B15] Lee B.-C. Kim J. Chen S. Sienko K. H. (2012). Cell phone based balance trainer. *J. Neuroeng. Rehabil.* 9:10. 10.1186/1743-0003-9-10 22316167 PMC3340298

[B16] Lindquist M. A. Mejia A. (2015). Zen and the art of multiple comparisons. *Psychosom. Med.* 77 114–125. 10.1097/psy.0000000000000148 25647751 PMC4333023

[B17] Mowinckel A. M. Espeseth T. Westlye L. T. (2012). Network-specific effects of age and in-scanner subject motion: a resting-state fMRI study of 238 healthy adults. *Neuroimage* 63 1364–1373. 10.1016/j.neuroimage.2012.08.004 22992492

[B18] Noohi F. Kinnaird C. De Dios Y. Kofman I. Wood S. J. Bloomberg J. J. (2019). Deactivation of somatosensory and visual cortices during vestibular stimulation is associated with older age and poorer balance. *PLoS One* 14:e0221954. 10.1371/journal.pone.0221954 31513630 PMC6742389

[B19] Noohi F. Kinnaird C. DeDios Y. Kofman I. S. Wood S. Bloomberg J. (2017). Functional brain activation in response to a clinical vestibular test correlates with balance. *Front. Syst. Neurosci.* 11:11. 10.3389/fnsys.2017.00011 28344549 PMC5344901

[B20] Peelle J. E. Troiani V. Wingfield A. Grossman M. (2010). Neural processing during older adults’ comprehension of spoken sentences: age differences in resource allocation and connectivity. *Cereb. Cortex* 20 773–782. 10.1093/cercor/bhp142 19666829 PMC2837088

[B21] Pleger B. Blankenburg F. Bestmann S. Ruff C. C. Wiech K. Stephan K. E. (2006). Repetitive transcranial magnetic stimulation-induced changes in sensorimotor coupling parallel improvements of somatosensation in humans. *J. Neurosci.* 26 1945–1952. 10.1523/jneurosci.4097-05.2006 16481426 PMC2635564

[B22] Reuter-Lorenz P. A. Cappell K. A. (2008). Neurocognitive aging and the compensation hypothesis. *Curr. Dir. Psychol. Sci.* 17 177–182. 10.1111/j.1467-8721.2008.00570.x

[B23] Roski C. Caspers S. Langner R. Laird A. R. Fox P. T. Zilles K. (2013). Adult age-dependent differences in resting-state connectivity within and between visual-attention and sensorimotor networks. *Front. Aging Neurosci.* 5:67. 10.3389/fnagi.2013.00067 24194718 PMC3810651

[B24] Seidler R. Erdeniz B. Koppelmans V. Hirsiger S. Mérillat S. Jäncke L. (2015). Associations between age, motor function, and resting state sensorimotor network connectivity in healthy older adults. *Neuroimage* 108 47–59. 10.1016/j.neuroimage.2014.12.023 25514517

[B25] Sienko K. H. Balkwill M. D. Oddsson L. I. E. Wall C. (2008). *Journal of Vestibular Research: Equilibrium and Orientation., Journal of Vestibular Research.* Amsterdam: Elsevier Science.

[B26] Tomasi D. Volkow N. D. (2012). Aging and functional brain networks. *Mol. Psychiatry* 17 549–558. 10.1038/mp.2011.81 21727896 PMC3193908

[B27] Whitfield-Gabrieli S. Nieto-Castanon A. (2014). *CONN – fMRI Functional Connectivity Toolbox BATCH Processing Manual. 81.* Cambridge, MA: McGovern Institute for Brain Research.

[B28] Yang Z. Chang C. Xu T. Jiang L. Handwerker D. A. Castellanos F. X. (2014). Connectivity trajectory across lifespan differentiates the precuneus from the default network. *Neuroimage* 89 45–56. 10.1016/j.neuroimage.2013.10.039 24287438 PMC3944140

[B29] Yuan J. Blumen H. M. Verghese J. Holtzer R. (2015). Functional connectivity associated with gait velocity during walking and walking-while-talking in aging: a resting-state fMRI study. *Hum. Brain Mapp.* 36 1484–1493. 10.1002/hbm.22717 25504964 PMC4373975

[B30] zu Eulenburg P. Caspers S. Roski C. Eickhoff S. B. (2012). Meta-analytical definition and functional connectivity of the human vestibular cortex. *Neuroimage* 60 162–169. 10.1016/j.neuroimage.2011.12.032 22209784

